# Dosing Regimen of Aditoprim and Sulfamethoxazole Combination for the *Glaesserella parasuis* Containing Resistance and Virulence Genes

**DOI:** 10.3390/pharmaceutics14102058

**Published:** 2022-09-27

**Authors:** Anxiong Huang, Xiao Huang, Zhihao Zhang, Zonghui Yuan, Lingli Huang, Yulian Wang, Yanfei Tao, Dongmei Chen, Zhenli Liu, Haihong Hao

**Affiliations:** 1 National Reference Laboratory of Veterinary Drug Residue, Huazhong Agricultural University, Wuhan 430070, China; 2 MOA Laboratory for Risk Assessment of Quality and Safety of Livestock and Poultry Products, Huazhong Agricultural University, Wuhan 430070, China; 3 Shenzhen Institute of Nutrition and Health, Huazhong Agricultural University, Shenzhen 518000, China; 4 Shenzhen Branch, Guangdong Laboratory for Lingnan Modern Agriculture, Shenzhen 518000, China; 5 Genome Analysis Laboratory of the Ministry of Agriculture, Agricultural Genomics Institute at Shenzhen, Chinese Academy of Agricultural Sciences, Shenzhen 518000, China

**Keywords:** *Glaesserella parasuis*, aditoprim, sulfamethoxazole, dosing regimen, genomic

## Abstract

*Glaesserella parasuis* (*G. parasuis*) causes Glasser’s disease in pigs and causes high mortality in piglets. The new drug Aditoprim (ADP) alone or combined with Sulfamethoxazole (SMZ) is one of the good choices for treating respiratory infections. The objective of this study was to recommend the optimal dosing regimen for the treatment of *G. parasuis* infection which contains resistance and virulence genes by ADP/SMZ compound through pharmacokinetics–pharmacodynamics (PK-PD) modeling. The whole genome of the virulent strain *G. parasuis* H78 was obtained and annotated by whole genome sequencing. The results show that *G. parasuis* H78 consists of a unilateral circular chromosome with prophages in the genome. The annotation results of *G. parasuis* H78 showed that the genome contained a large number of virulence-related genes and drug resistance-related genes. The in vitro PD study showed that the antibacterial effect of ADP/SMZ compound against *G. parasuis* was time-dependent, and AUC/MIC was selected as the PK-PD modeling parameter. The PK study showed that the content of ADP/SMZ compound in pulmonary epithelial lining fluid (PELF) was higher than plasma, and there were no significant differences in ADP and SMZ PK parameters between the healthy and infected group. The dose equation to calculate the optimal dosing regimen of ADP/SMZ compound administration for control of *G. parasuis* infection was 5/25 mg/kg b.w., intramuscular injection once a day for 3~5 consecutive days. The results of this study provide novel therapeutic options for the treatment of *G. parasuis* infection to decrease the prevalence and disease burden caused by *G. parasuis*.

## 1. Introduction

*Glaesserella parasuis* (*G. parasuis*) is a common pathogen in the respiratory tract of conventional pig farms. It can infect pigs and cause serious systemic diseases, also known as Glasser’s disease, characterized by fibrinous polyserositis, pleurisy, arthritis, and meningitis [1]. The early clinical symptoms of pigs after infection are dyspnea, joint swelling, lameness, skin cyanosis, etc., and death 2~3 days after the onset of the disease [2], causing serious economic losses [3]. *G. parasuis* has many virulent serotypes, such as serotypes 4, 5, 10, 12, 13, and 14, and serotypes 4 and 5 are dominant in China [4,5,6]. There are many antibiotics for the treatment of swine respiratory diseases. At present, the more commonly used antibiotics are oxytetracycline hydrochloride, enrofloxacin, tiamulin, tylosin, kanamycin, lincomycin, etc. [7,8,9]. However, due to the serious development of antibiotic resistance, many commonly used antibiotics have no effect on *G. parasuis*. Therefore, it is necessary to find new antibiotics or combined antibiotics for the clinical treatment of *G. parasuis* infection.

Early studies have shown that sulfamethoxazole (SMZ) has a broad antibacterial spectrum against *Streptococcus*, *Pneumococcus*, *Salmonella*, *Pasteurella*, *Clostridium perfringens*, etc. [10,11,12,13]. However, recent research reports that SMZ gradually shows universal resistance to the above bacteria [14,15]. For *G. parasuis*, sulfonamides also show certain resistance [16]. At the same time, long-term use of SMZ can cause toxic effects, such as kidney damage, hematuria, crystalluria, allergic reactions, and even shock. Based on the increasing resistance of SMZ to most common bacteria, and to reduce the toxic effect of SMZ on animals when used in large quantities alone, SMZ is often combined with sulfonamide synergists in clinical treatment to double block the metabolism of bacteria and remove the original bacteria, transforming the antibacterial effect to a bactericidal effect, thereby enhancing the clinical treatment effect [17,18].

Aditoprim (ADP) is a new type of animal-specific sulfonamide synergist; it has a broad antibacterial spectrum and it is more sensitive to most bacteria than another sulfonamide synergist, trimethoprim (TMP) [19,20,21]. Its antibacterial ability when used in combination with sulfonamides is better than used with sulfonamides alone, indicating that the combined use has a synergistic antibacterial effect [22]. Toxicology studies have shown that ADP has low toxicity to animals, no genetic toxicity, and teratogenicity [23,24]. PK studies have shown that ADP has the characteristics of wide distribution in the body, large apparent volume, the long half-life, high bioavailability, etc. It can be used as an alternative to TMP and has broad market prospects [22,25,26,27,28].

The virulence of different *G. parasuis* isolates varies, ranging from subclinical carriers to severe systemic diseases, depending on the virulence factors carried by the isolates. The potential virulence factors of *G. parasuis* currently mainly include trimeric autotransporter (VtaA) [29,30], outer membrane proteins (OMPs) [31,32,33], polysaccharide biosynthesis protein (CapD) [34], regulatory proteins (QseC and OxyR) [35,36], ABC-type transporters (OppA, YfeA, and PlpA), and curli protein assembly (CsgG) [37]. In addition, capsular polysaccharide biosynthesis genes are also potential virulence factors of *G. parasuis* [38]. The strength of virulence is closely related to pathogenicity, and a good therapeutic effect on highly virulent strains will greatly improve the effectiveness of drugs in clinical practice. Similarly, different strains have different drug resistance backgrounds, and a good therapeutic effect on drug-resistant strains can improve the cure rate in clinical use.

In this paper, the virulence factors of the virulent strain *G. parasuis* H78 were revealed through whole genome sequencing, and a large number of drug resistance genes were found in the genome of the virulent strain *G. parasuis* H78. By studying the in vitro, ex vivo PD, and in vivo PK of the ADP, SMZ, and ADP/SMZ compound, the optimal dosing regimen of ADP/SMZ compound for the treatment of *G. parasuis* infection has been determined. These studies aim to provide a theoretical basis for the PK-PD of ADP/SMZ compound dose selection, as well as contribute to the control of the occurrence and development of drug resistance.

## 2. Materials and Methods

### 2.1. Chemicals and Reagents

Aditoprim (ADP, 99.0%) was made at Veterinary Drug Research Center, Huazhong Agricultural University, Wuhan, China. Sulfamethoxazole (SMZ, 98.0%) and chloramphenicol (99.0%) were purchased from the China Institute of Veterinary Drug Control. All the chemical reagents and organic solvents used were of analytical grade or higher grade.

### 2.2. Strains and Animals

A total of 104 *G. parasuis* strains were donated by the State Key Laboratory of Agricultural Microbiology of Huazhong Agricultural University and preserved by the National Veterinary Drug Residue Reference Laboratory. All of the bacteria Isolates were confirmed by polymerase chain reaction (PCR) of 16S rRNA [39]. *E. coli* (ATCC 25922) was used as the quality control strain.

Twenty-four eight-week-old healthy crossbred pigs (20 ± 2 kg) were purchased from Huazhong Agricultural University pig breeding farm. These pigs were put in separate houses with free access to the water and feed. For proper adaptation, these animals were fed with this feed for 1 week before the trial. Two hundred and six healthy Balb/c mice of similar weight (20 ± 2 g) were purchased from the Experimental Animal Center of Huazhong Agricultural University. Before experiments, mice were raised for 1 week to acclimatize. The research was approved by the Animal Ethics Committee of Huazhong Agricultural University (HZAUSW-2019-024). All procedures regarding animal care and testing were carried out according to the recommendation for the care and use of laboratory animals of Hubei provincial public service facilities.

### 2.3. Antimicrobial Susceptibility Monitoring

The in vitro PD study ratio of ADP/SMZ compound was determined to be 1:5 through the checkerboard technique of drug sensitivity test, preparation ratio, and drug compatibility.

The MICs of 104 strains of *G. parasuis* against ADP, SMZ, and ADP/SMZ compound were determined concerning the agar dilution method recommended by CLSI M07-A9 [40]. *E. coli* (ATCC 25922) and chloramphenicol were used as quality control, and the experimental results were only valid when the MIC was within the quality control range.

### 2.4. Screening of Highly Virulent Strains

The serotypes of *G. parasuis* were determined by Enterobacterial Repetitive Intergenic Consensus—PCR [41], and the most virulent serotype 5 *G. parasuis* was selected for mouse toxicity test.

The selected 13 strains of *G. parasuis* with serotype 5 were subjected to a mouse toxicity test. The lethality of *G. parasuis* in 126 healthy Balb/c mice was observed by intraperitoneal injection of different concentrations (10^9^ CFU/mL, 10^8^ CFU/mL, and 10^7^ CFU/mL) of bacterial solution. The mice were divided into 13 experimental groups and 1 control group for each high, middle, and low dose group. Each group had 3 mice. After injection, the mortality and death time of each mouse were observed and counted within 1 week.

The strains with lethality that reached 100% were tested for LD50 by mouse toxicity test. The lethality of *G. parasuis* in 80 healthy Balb/c mice was observed by intraperitoneal injection of different concentrations (1 × 10^8^ CFU/mL, 2 × 10^8^ CFU/mL, 5 × 10^8^ CFU/mL, and 1 × 10^9^ CFU/mL) of bacterial solution. The mice were divided into 5 experimental groups and 1 control group for each dose group. Each group had 4 mice. After injection, the mortality and death time of each mouse were observed and counted within 24 h.

The most virulent strains were selected for whole-genome sequencing analysis and PK-PD studies.

### 2.5. Genome Sequencing and Bioinformatics Analysis

Genomes of the virulent strain *G. parasuis* H78 with serotype 5 were sequenced. In this project, the third-generation sequencer PacBio RSII [42] was used to sequence *G. parasuis* H78, and the genome was assembled through the HGAP3 process [43] after obtaining the original DNA sequencing data. After the assembly is completed, the original data are aligned to the assembled reference genome sequence, and the coverage depth of each position of the reference genome sequence is calculated to evaluate the integrity of the genome assembly and sequencing uniformity. The genome was predicted to encode gene structure by Prodigal v2.6.3 software [44], and tRNA and rRNA were predicted by tRNAscan-SE v1.3.1 [45] and rRNAmmer v1.2 [46] software, respectively. Gene islands were mainly predicted by IslandPath-DIOMB v0.2 software [47], transposons were mainly predicted by TransposonPSI analysis (http://transposonpsi.sourceforge.net/, accessed on 13 August 2019), and prophages were predicted by PHAST analysis [48]. CRISPR prediction was performed on the sample genome by CRISPRFinder [49].

### 2.6. Gene Function Annotation

Gene function annotation is mainly based on amino acid sequence alignment. The protein sequences were aligned with each database based on BLAST (blastp, evalue ≤ 1 × 10^−5^) to obtain the corresponding functional annotation information. Since each sequence may have multiple alignment results, to ensure its accuracy, the result with the best alignment score is selected as the final annotation of this gene. All annotations were completed using BLAST software combined with the characteristics of each database. The databases used in this study were KEGG (Kyoto Encyclopedia of Genes and Genomes) [50], COG (Cluster of Orthologous Groups of proteins) [51], GO (Gene Ontology) [52], NR (Non-Redundant Protein Database) [53], Swissprot [54], Trembl [55], and Pfam [56] (Supplementary Table S1).

### 2.7. In Vitro and Ex Vivo Pharmacodynamics

The MIC and MBC of *G. parasuis* H78 in broth and pulmonary epithelial lining fluid (PELF) were determined by the broth dilution method according to the CLSI M07-A9 standard with some modifications. The MPC of *G. parasuis* H78 in broth was determined by the agar dilution method recommended by CLSI M07-A9. The post-antimicrobial effect (PAE) was determined by the method of bacterial and drug removal after incubation.

The in vitro and ex vivo time killing curves of ADP and ADP/SMZ compound in broth and PELF were drawn by monitoring the colony-formed unit (CFU) changes during the incubation of *G. parasuis* H78 under a series of concentrations of ADP and ADP/SMZ compound for a continuous time points (0, 1, 2, 4, 6, 8, 12, and 24 h).

### 2.8. Pharmacokinetics Animal Experiment Design

Twenty-four pigs were randomly divided into 4 groups with 3 healthy groups and 1 infected group. The 3 healthy groups were given a single intramuscular injection of ADP (5 mg/kg b.w.), SMZ (25 mg/kg b.w.), and ADP/SMZ compound (5/25 mg/kg b.w.). The infected group was infected with *G. parasuis* H78 for 3 days. After the successful establishment of the infected model, a single intramuscular injection of ADP/SMZ compound (5/25 mg/kg b.w.) was produced. The plasma and PELF of each group were collected at different time points (0.5, 1, 2, 3, 4, 6, 8, 12, 24, 36, and 48 h) after administration.

### 2.9. The HPLC Method of Determine ADP and SMZ in Plasma and PELF

Extraction of ADP and SMZ in plasma and PELF: Accurately pipette 1 mL of plasma or PELF into a 10 mL centrifuge tube. Add 3 mL of a mixture of dichloromethane and acetonitrile, and vortex centrifuge for 5 min at 10,000 rpm for 10 min. Remove the supernatant into a 10 mL centrifuge tube. Add 3 mL of dichloromethane and acetonitrile to the residue. Extract once again as described above. Combine the supernatant twice, and dissolve the residue with 1 mL of the initial mobile phase. Vortex to mix. Add 1 mL of n-hexane, remove the n-hexane layer (upper layer) to achieve the purpose of degreasing, and then use a 0.22-μm filter membrane to obtain a sample purification solution.

The concentration of ADP and SMZ in the extracted plasma and PELF was detected by high performance liquid chromatography (HPLC). A C18 reverse phase column (250 mm × 4.6 mm, i.d., 5 µm, Agilent, Santa Clara, CA, USA) was used for HPLC, which was executed at a detection wavelength of 270 nm at 25 °C. The mobile phase comprised 0.1% trifluoroacetic acid (phase A) and 100% methanol (phase C) with a mobile phase flow rate of 1 mL/min.

### 2.10. Quantitation of PELF Volume

The urea dilution method was used to determine the volume of PELF as described previously [57,58]. The concentration of urea and PELF were determined by the urease-glutamate dehydrogenase enzymatic method with an automatic biochemical analyzer (SYNCHRON CX4 PRO; Beckman, Brea, CA, USA) at the National Reference Laboratory of Veterinary Drug Residues (Wuhan, China).

### 2.11. Establishment of PK-PD Modeling

The PK-PD (*AUC*_24h_/*MIC*) target was calculated based on the inhibitory effect (*E*) of the ADP/SMZ compound against *G. parasuis* H78 growth obtained from the ex vivo time killing curve using the sigmoid *E_max_* model in WinNonlin. The model was described by the Hill formulation: $E=E_{max}-\frac{(E_{max}-E_{0})\cdot C^{N}}{C^{N}+EC_{50}^{N}}$, where *E* represents the effect of the antimicrobial agent calculated as the log10 difference in bacterial number before and after 24 h incubation in vitro. The *EC*_50_ value indicates the *AUC*_0−24h_/*MIC* value reached 50% of *E_max_*, *C* represents the *AUC*_0−24h_/*MIC* ratio, and *N* shows the Hill coefficient.

### 2.12. Optimization of Dosing Regimen

The dose equation: $\mathrm{Dose}=\frac{\mathrm{CL}\times {\left(\mathrm{AUC}/\mathrm{MIC}\right)}_{\mathrm{BP}}\times \mathrm{MIC}}{F\times \mathrm{fu}}$, was used to calculate the dose of ADP and SMZ for treatment of *G. parasuis,* where *CL* referred to the clearance per day of ADP and SMZ in swine; (*AUC*/*MIC*)*_BP_* referred to the PK-PD target; *F*, bioavailability; *fu*, free ratio of the drug. To obtain the free ratio of the drug (*fu*), the binding rate of ADP and SMZ in PELF was determined by equilibrium dialysis and the HPLC method.

## 3. Results

### 3.1. MIC Distribution

**Figure 1:**
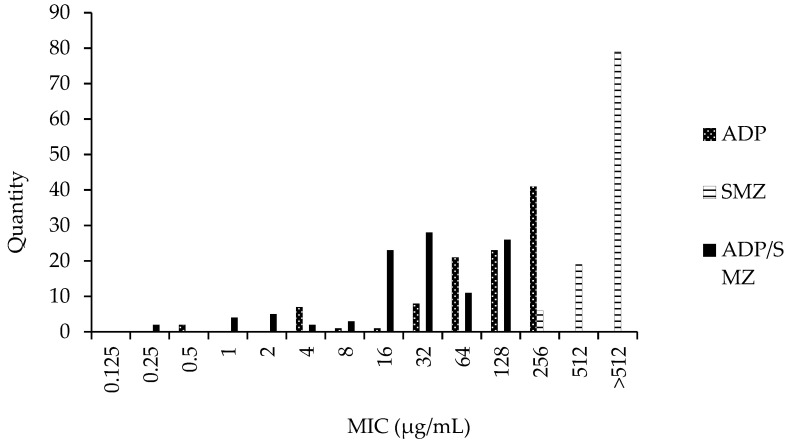
The MIC distribution of ADP, SMZ, and ADP/SMZ (MIC to ADP concentration) against 104 *G. parasuis*.

All *G. parasuis* isolates were confirmed by a PCR-based method. The minimum inhibitory concentration (MIC) of chloramphenicol (China Institute of Veterinary Drug Control) against *E.* *coli* (ATCC 25922) was 4 µg/mL, which is within the quality control range. The MIC of ADP, SMZ, and ADP/SMZ compound against 104 *G. parasuis* strains was determined by the agar dilution method following the CLSI M07-A9 standard, and the MIC result is shown in Figure 1. It can be seen from Figure 1 that the effect of the ADP/SMZ compound is better than that of a single ADP and SMZ.

### 3.2. Mouse Toxicity Test Results

The results of the mouse toxicity test (Table 1) showed that in the high-dose group, *G. parasuis* H78 was the most pathogenic, and all mice died within 24 h. In the middle-dose group, *G. parasuis* H78 was the most pathogenic, with 33.3% in one week of lethality. No mice died in the low-dose group. At the same time, in the acute LD50 experiment, *G. parasuis* H78 required the lowest amount of bacteria to reach LD50 within 24 h. Therefore, *G. parasuis* H78 was selected for whole-genome sequencing analysis and PK-PD study.

### 3.3. Genome Features and Genome Component of G. parasuis H78

The complete genome assembly and annotation of *G. parasuis* H78 contains unilateral circular chromosomes of 2,315,317 base pairs (bp) in length. The sequencing depth was 377X and the result was of good integrity. The genomes of *G. parasuis* H78 encode 3109 genes with G+C contents of 39.89%, and the percentage of coding gene sequence lengths in the total genome was 84.67%. The number of rRNAs and tRNAs in the *G. parasuis* H78 genomes was 19 and 58, respectively. The number of gene islands, transposons, and CRISPRs in the *G. parasuis* H78 genomes was 7, 1, and 7, respectively.

Using PHAST prediction to assess prophage regions in the genomes of *G. parasuis* H78, it was found that nine prophage regions were identified on the *G. parasuis* H78 chromosome, of which five were complete and four were incomplete. The identified prophage regions are unique to each genome. Table 2 summarizes the chromosomal locations of the prophages of the *G. parasuis* H78, including starting and ending nucleotide positions, phage region length, phage region classification, and phage keywords.

**Figure 2:**
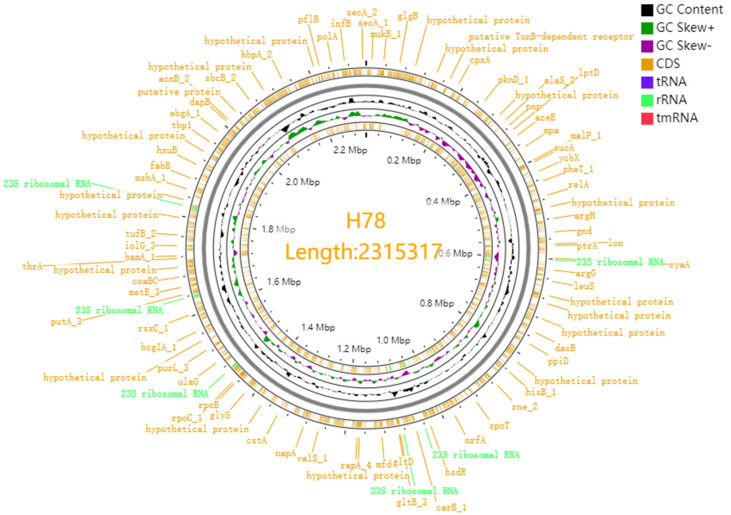
Genetic map of *G. parasuis* H78.

The genomes of *G. parasuis* H78 were submitted to the Proksee system, and after genome assembly and annotation, a visual gene map was drawn (Figure 2).

### 3.4. Virulence-Associated Genes Identified in G. parasuis H78

To identify factors that might support host colonization and/or virulence, the *G. parasuis* H78 annotations were searched for genes encoding predicted functions for adhesion, hemolysis, secretion, toxin production, or other virulence-related effects (Supplementary Table S2).

A total of 21 genes belong to 14 types encoding predicted adhesins were identified in *G. parasuis* H78, including four outer membrane proteins (OmpA, OmpP1, OmpP2, and OmpP5), four pilus assembly proteins (PilA, PilB, PilC, and PilD), pertactin family virulence factor AidA, Metal transporter substrate-binding protein ZnuA, Uncharacterized lipoprotein NlpE, PilT domain-containing protein, and two type IV fimbriae genes.

A total of 13 genes belonging to six types encoding predicted hemolysins were identified in *G. parasuis* H78, including Osmotically-inducible protein OsmY, Hemolysin secretion protein D, Hemolysin regulation protein AhpA, Hemolysin activation/secretion protein ShlB, and two unnamed Hemolysin related protein.

A total of 9 genes belonging to six types encoding predicted secretion were identified in *G. parasuis* H78, including Sec-independent protein secretion pathway component TatC, Tat protein secretion system quality control protein TatD, Curli biogenesis system outer membrane secretion channel CsgG, 2 Type II secretory pathway, and 1 Type I secretion system permease/ATPase.

*G. parasuis* H78 contains a large number of toxin-antitoxin (TA) systems. The TA system is a small genetic element composed of two components, a stable protein toxin and an unstable antagonistic antitoxin, which can be a protein or noncoding RNA [59]. The bacterial TA system is not only associated with bacterial pathogenicity [60], but also affects biofilm formation and persister cell formation [61], and plays an important role in multidrug resistance [62]. According to the type of antitoxin (RNA or protein) and mode of action, TA systems are classified into six types (types I–VI) [60]. Among them, the type II TA system is the most abundant in prokaryotes [59]. It is a small protein encoded by a gene in a bicistronic operon, and the antitoxin blocks the toxicity of the toxin by forming a complex with the toxin [61,62,63]. *G. parasuis* H78 contains several type II TA families, including RelBE, MazEF, and HigBA, the most abundant of which are the six putative higBA loci identified in *G. parasuis* H78.

### 3.5. Resistance-Associated Genes Identified in G. parasuis H78

To identify factors that may influence antibiotic resistance, *G. parasuis* H78 annotations were searched for genes encoding predictive functions for antibiotic resistance-related effects. A total of 20 antibiotic resistance genes belonging to nine types were found in *G. parasuis* H78, most of which were associated with multidrug resistance (Supplementary Table S3).

In addition, some genes related to the metal ion and amino acid resistance were also found in *G. parasuis* H78.

### 3.6. In Vitro and Ex Vivo PD Study of G. parasuis H78

The MIC of ADP and ADP/SMZ compound against *G. parasuis* H78 in vitro was 4 μg/mL and 2/10 μg/mL, respectively; and the minimum bactericidal concentration (MBC) in vitro was 8 μg/mL and 4/20 μg/mL, respectively. The mutant prevention concentration (MPC) of ADP against *G. parasuis* H78 was 20 μg/mL. When the culture was in PELF, the MIC of ADP/SMZ compound against *G. parasuis* H78 ex vivo was 0.5/2.5 μg/mL, and the MBC ex vivo was 1/5 μg/mL.

The post-antimicrobial effect (PAE) of ADP and ADP/SMZ against *G. parasuis* H78 are shown in Table 3. The PAE of *G. parasuis* exposed to different concentrations of ADP/SMZ compound showed a positive correlation with the drug concentration and exposure time. The higher the drug concentration, the greater the PAE, and the longer the exposure time, the greater the PAE. However, the PAE of the ADP/SMZ combination was significantly higher than that of ADP alone, indicating that there was a synergistic effect between the two drugs.

### 3.7. In Vitro and Ex Vivo Time Killing Curves

**Figure 3:**
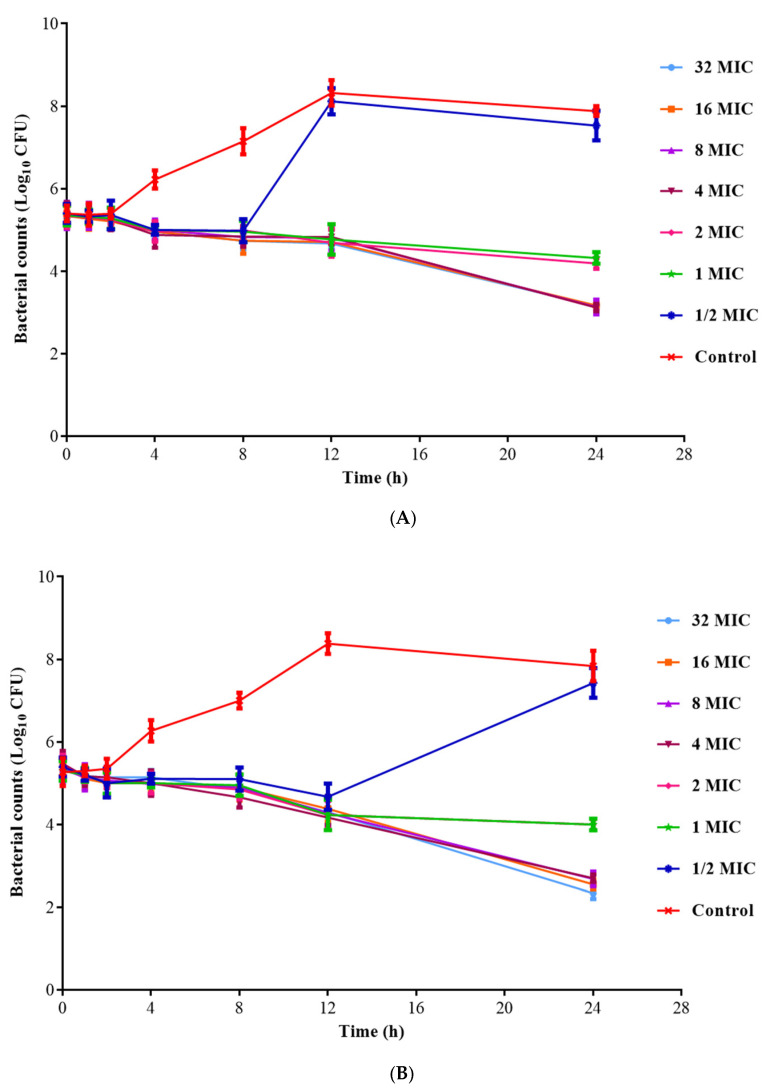
The time killing curves of ADP (**A**) and ADP/SMZ compound (**B**) against *G. parasuis* H78 in broth.

The in vitro time killing curves of ADP and ADP/SMZ compound against *G. parasuis* H78 in broth are shown in Figure 3. As displayed in Figure 3, the in vitro bactericidal effect of ADP and ADP/SMZ compound against *G. parasuis* were similar. When the concentrations of ADP and ADP/SMZ compound were higher than 4MIC, the antibacterial efficiency reaches saturation. Therefore, the in vitro time killing curve shows that the activity of ADP and ADP/SMZ compound against *G. parasuis* was time-dependent, consistent with reported research [64].

**Figure 4:**
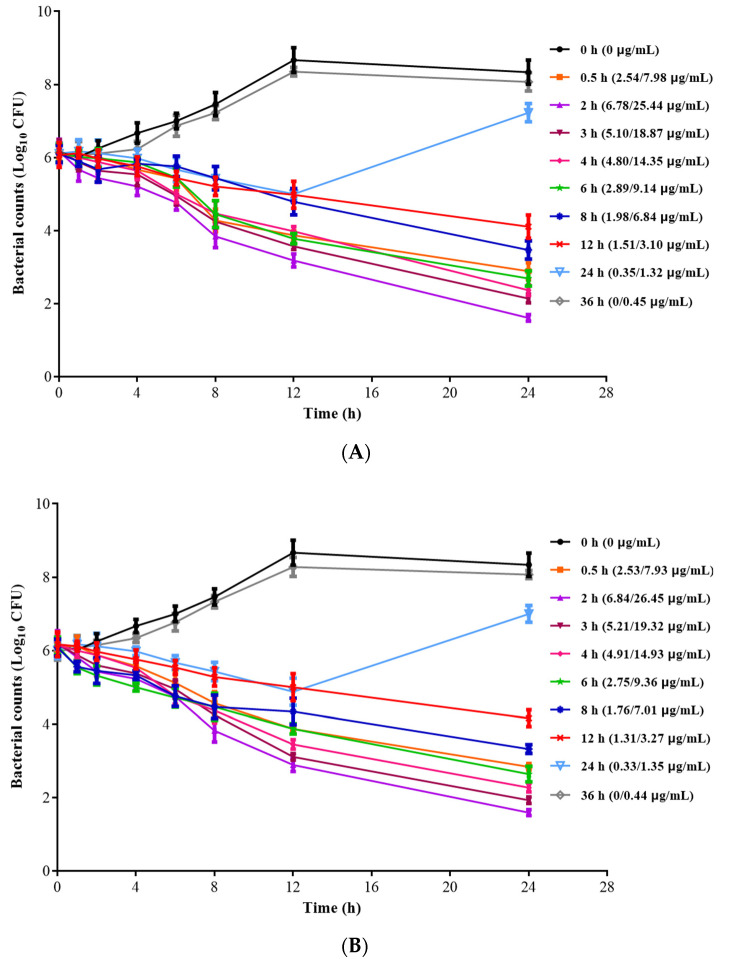
The time killing curves of ADP/SMZ compound against *G. parasuis* H78 in healthy (**A**) and infected (**B**) pigs PELF.

The ex vivo time killing curves of ADP/SMZ compound against *G. parasuis* H78 in PELF of the healthy and infected groups are shown in Figure 4. The results showed that the PELF samples collected within 12 h in the healthy and infected groups could inhibit the growth of bacteria. The ADP/SMZ compound in the PELF samples collected at 0.5~12 h after the administration had an obvious bactericidal effect. The higher concentration of ADP and SMZ in the lung contributed to the stronger bactericidal activity. The type of antimicrobial activity was time-dependent, which was the same as the in vitro PD study above. Therefore, the AUC/MIC was selected as the PK-PD parameter.

### 3.8. The PK Study of ADP and ADP/SMZ in Healthy and Infected Pigs

The limit of determination (LOD) and the limit of quantification (LOQ) of ADP and SMZ both in plasma and PELF were 0.05 µg/mL and 0.1 µg/mL, respectively. This method also had a high precision (ADP inter-day RSD < 6.38% and SMZ inter-day RSD < 6.62%), ADP recovery (80.1–101.3%) and SMZ recovery (87.6–97.2%) in plasma, ADP recovery (84.0–94.4%) and SMZ recovery (82.8–101.5%) in PELF. A standard curve was established: y = 21,227x + 68.65 (ADP in plasma), y = 59,316x − 604.7 (SMZ in plasma), y = 21,460x + 19.03 (ADP in plasma) and y = 58,815x + 2134.2 (SMZ in PELF).

**Figure 5:**
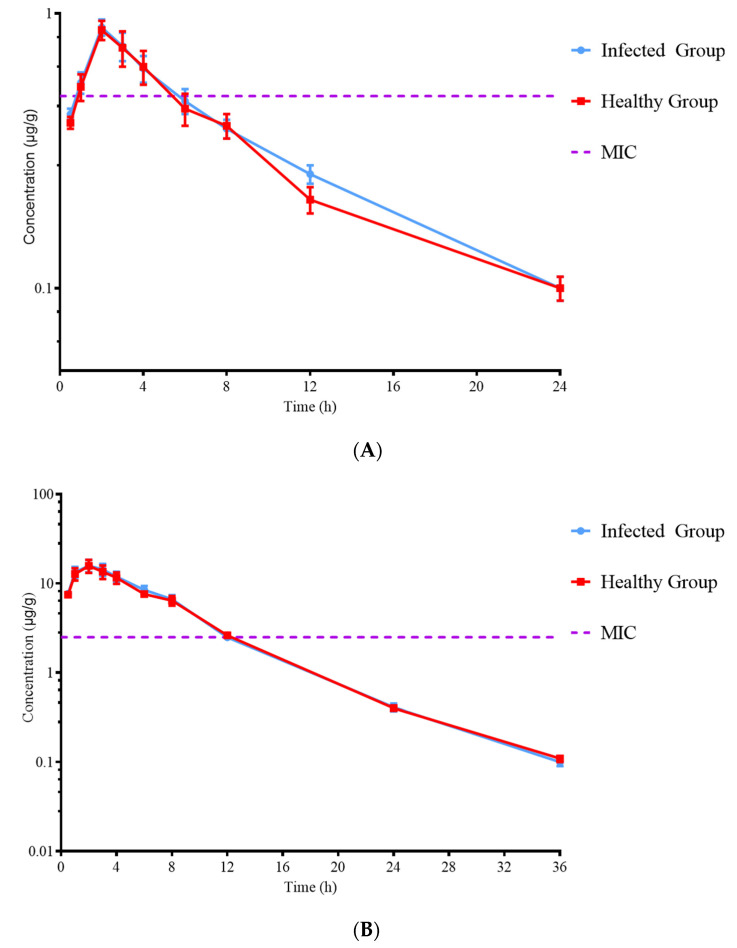
Concentrations of ADP (**A**) and SMZ (**B**) in plasma at various time points.

**Figure 6:**
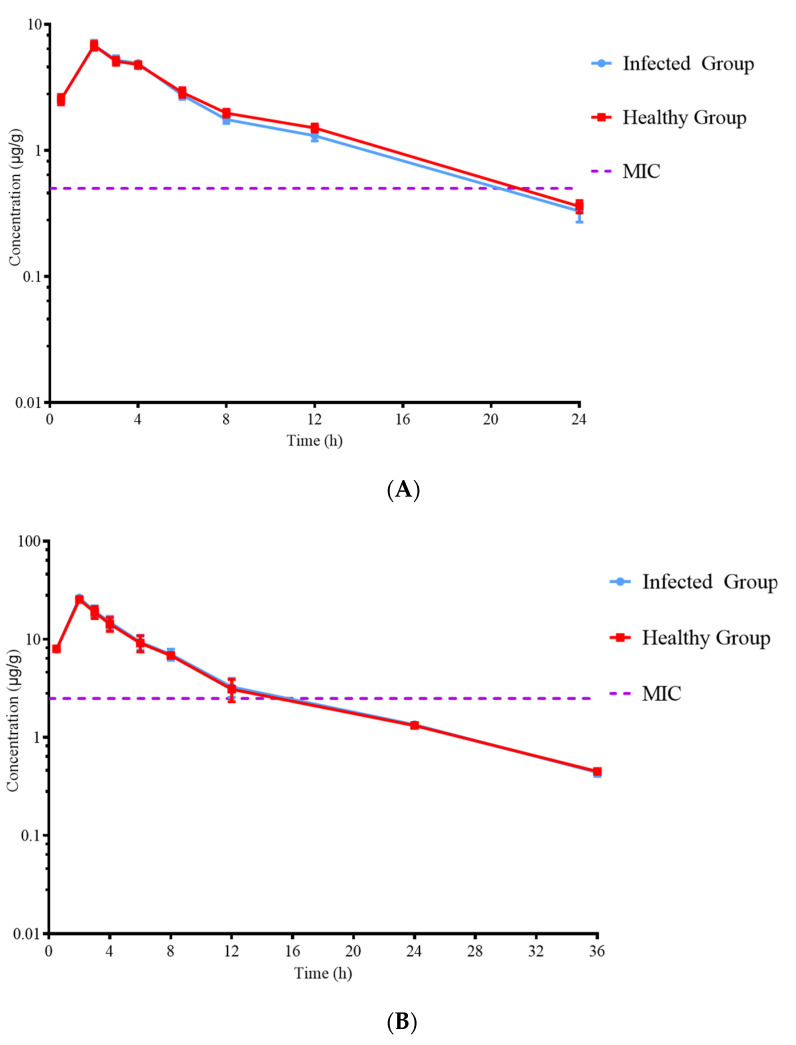
Concentrations of ADP (**A**) and SMZ (**B**) in PELF at various time points.

No serious adverse reactions occurred after intramuscular injection of ADP, SMZ, and ADP/SMZ compound. A single dose of 5 mg/kg b.w., 25 mg/kg b.w., and 5/25 mg/kg b.w. ADP, SMZ, and ADP/SMZ compound, respectively, was administered. ADP and SMZ in plasma were less than PELF by the HPLC method. The concentration–time curves of ADP/SMZ compound in plasma and PELF of the healthy and infected groups are shown in Figure 5 and Figure 6.

After 24 h, the concentration of ADP and SMZ in plasma decreased below LOD, and after 36 h, the concentration of ADP and SMZ in PELF decreased below LOD. The fitting time of the model was 0–24 h, and the non-compartment model was used. The parameters of ADP, SMZ, and ADP/SMZ compound in plasma and PELF were simulated with WinNonlin (v.5.2.1 US Certara Pharsight^®^). The parameters are shown in Table 4, Table 5 and Table 6.

### 3.9. PK-PD Modeling Integration

The association between antimicrobial efficiency and the ex vivo PK-PD parameters of *AUC*_0−24h_/*MIC* ratios was simulated by using the model of *E_max_* inhibitory Sigmoid. The parameters of the Hill coefficient *N*, *E*_0_, *E_max_*, and *AUC*_0–24h_/*MIC* values of the model for three levels of the growth inhibition are shown in Table 7.

### 3.10. Dosing Regimen for the Clinical Study

Based on the dose equation and Monte Carlo simulation, the daily doses of ADP and SMZ for healthy and infected required to predict the bactericidal, bacteriostatic, and elimination activities of *G. parasuis* H78 are shown in Table 8. Considering the dosage of healthy and infected under treatment conditions and the drug ratio, the best dosing regimen was ADP/SMZ compound 5/25 mg/kg b.w., once a day for 3–5 days.

## 4. Discussion

*G. parasuis* is mainly responsible for Glasser’s disease, which is characterized by polyserositis, arthritis, and meningitis [7,65,66]. The outbreak and widespread infection caused by *G. parasuis* bring serious harm and economic loss to intensive and large-scale aquaculture worldwide. The emergency resistance of *G. parasuis* to some veterinary critical important antimicrobial agents, especially the multiple drug resistance has threatened clinical treatment and disease control. After reviewing many papers and summarizing the antibiotic resistance of *G. parasuis* in the past 10 years, it was found that the drug resistance rate to tetracycline, sulfa, fluoroquinolones, and other drugs is high, up to 80% and above [67,68,69]. To solve this problem, the combined use of antibiotics is now also advocated because of their clinical effects, e.g., TMP/SMZ, including the ones mentioned in this article on ADP/SMZ compound [70].

Whole genome sequencing is a method to study and analyze the structural differences between different individual genomes using biological information, and to complete SNP and genome structure annotation at the same time. In this study, the whole genomes of the virulent strains *G. parasuis* H78 were sequenced, and the sequencing results were similar to those of the virulent strains *G. parasuis* Nagasaki, both of which had a unilateral circular chromosomes composition, and the whole genome length and CG% were also similar [71]. However, there is a big difference in the number of CDSs. The virulent strain *G. parasuis* Nagasaki has 2173 functional CDSs, and *G. parasuis* H78 has 3109 coding genes, respectively, which can be related to the annotation database used.

The bactericidal characteristic of an antibiotic is closely dependent on the organism–drug combination. The same drug is likely to show distinct bactericidal features to different bacterial strains. Since ADP/SMZ compound antibiotic is a newly synthetic antibiotic, there is no paper report on the type of sterilization. However, it can be compared with the same drug TMP/SMZ. In the study of TMP/SMZ, it is found that there is both time-dependent and concentration-dependent tolerance, which may be related to the type of bacteria [64,72]. The ADP/SMZ compound has a good antibacterial effect on the *G. parasuis* separated from the infected pigs. In vitro and ex vivo time killing curves showed that the type of action of ADP/SMZ compound against *G. parasuis* of pigs was both time-dependent and concentration-dependent. When the concentration of ADP and ADP/SMZ compound is higher than the MIC of the strain, as the concentration of the drug increases, the antibacterial effect gradually increases, showing a certain concentration-dependence. However, when the concentration is greater than 4MIC, the antibacterial effect reaches saturation, showing a time-dependence.

The PK of ADP and SMZ single in the plasma of cattle, sheep, swine, and chickens have been previously described. At present, the most powerful and commonly used sulfonamides are TMP/SMZ compound injections. However, there are some problems with these compound injections, e.g., the biological half-life in animals is relatively short and cannot achieve the long-term antibacterial effect. Compared with TMP, the elimination half-life of ADP was longer than 2.25 h of TMP in six-month-old castrated boar, and the apparent distribution volume was also larger than 1.4 L/kg of TMP, indicating that ADP was more widely distributed in vivo [22]. This makes ADP have a better effect on the deep organization in the use of combined SMZ.

PK-PD research is a common method to determine the dosage regimen, and it is mostly used to formulate the optimal dosage regimen for a drug. There are few studies on formulating a combined dosing regimen of two or more drugs, and most of them are in the direction of human medicine. Cheng established the optimal dosage of TMP/SMZ for the treatment of melioidosis in humans through PK-PD research [72]. Alsaad established the optimal dosage of TMP/SMZ for the treatment of human Mycobacterium tuberculosis infection through PK-PD research [73,74]. It has also been reported that TMP/SMZ is used in the treatment of bacterial infections of aquatic organisms, e.g., *Portunus trituberculatus* [75]. Wang studied the pharmacokinetics of ADP/SMZ in porcine plasma and ileum contents. The results were similar to this study, where the optimal dosage of ADP/SMZ compound in the treatment of porcine *E. coli* infection was formulated as 3.45/17.25 mg/kg b.w., intramuscular injection every day for three consecutive days [76].

## 5. Conclusions

In this study, the drug susceptibility test of 104 strains of *G. sparasuis* to ADP/SMZ compound was performed, and by comparison with the sensitivity test of two single drugs, it was found that the combined use can effectively improve the efficacy. The two drugs have a synergistic effect after mixing in a ratio of 1:5, and the effect was more than 10 times that of the control group. A highly virulent *G. parasuis* H78 was screened for ex vivo PD and in vivo PK studies, and the virulence factors and drug resistance genes of *G. parasuis* H78 were found by whole genome sequencing. Finally, the PK–PD integration model data were obtained by fitting ex vivo PD and in vivo PK data. Based on the analysis and comparison of these data, the final reasonable dosage of ADP/SMZ compound was 5/25 mg/kg b.w., intramuscular injection once a day for three consecutive days, which represents a new and effective treatment in the clinic.

## Figures and Tables

**Table 1:** The lethality and LD50 of different *G. parasuis* in mice.

*G. parasuis* Strains	Infectious Dose			LD50/24 h (CFU/mL)
	10^9^ CFU/mL	10^8^ CFU/mL	10^7^ CFU/mL	
H2	0	0	0	-
H10	100%	0	0	1 × 10^9^
H12	33.3%	0	0	-
H17	0	0	0	-
H25	100%	0	0	1 × 10^9^
H27	100%	0	0	8 × 10^8^
H32	100%	0	0	8 × 10^8^
H42	66.7%	0	0	-
H52	66.7%	0	0	-
H62	66.7%	0	0	-
H78	100%	33.3%	0	5 × 10^8^
H1005	33.3%	0	0	-
H1074	0	0	0	-

**Table 2:** Prophage regions identified in *G. parasuis* H78.

Strain	Region	Region_Position	Length	Classification	Specific_Keyword
H78	1	62,800–103,517	40,718	intact	integrase, transposase, terminase
	2	200,059–212,818	12,760	incomplete	protease
	3	208,615–267,138	58,524	intact	terminase, lysin, recombinase, integrase
	4	361,139–369,931	8793	incomplete	transposase, protease
	5	784,187–817,679	33,493	intact	lysin, terminase
	6	1,087,501–1,122,214	34,714	intact	integrase, capsid, terminase
	7	1,762,009–1,776,053	14,045	intact	transposase
	8	2,037,372–2,048,976	11,605	incomplete	NA
	9	2,271,263–2,280,833	9571	incomplete	transposase

**Table 3:** The PAE of ADP and ADP/SMZ against *G. parasuis* H78.

Concentration (μg/mL)	ADP (h)		ADP/SMZ (h)	
	1 h	2 h	1 h	2 h
1 × MIC	0.02	0.40	0.27	0.46
2 × MIC	0.11	0.43	0.34	0.49
4 × MIC	0.20	0.48	0.63	1.11

**Table 4:** The PK parameters of ADP and SMZ in plasma after intramuscular injection of ADP/SMZ injection at 5/25 mg/kg b.w. of SMZ in swine (*n* = 6).

Parameters	Unite	Healthy Group		Infected Group	
		ADP	SMZ	ADP	SMZ
*AUC*_24h_	h·μg/mL	7.71 ± 0.26	120.70 ± 10.24	8.00 ± 0.45	124.29 ± 7.11
*MRT*_24h_	h	7.76 ± 0.26	6.55 ± 0.12	7.85 ± 0.09	6.48 ± 0.15
*T*_1/2λ_	h	7.97 ± 0.81	4.68 ± 0.2	8.45 ± 0.61	5.00 ± 0.38
*CL*/*F*	mL/h/Kg	0.56 ± 0.03	0.21 ± 0.02	0.54 ± 0.04	0.20 ± 0.01
*T_max_*	h	2.00 ± 0.00	2.00 ± 0.00	2.00 ± 0.00	2.00 ± 0.00
*C_max_*	μg/mL	0.87 ± 0.09	15.72 ± 1.74	0.88 ± 0.05	16.01 ± 1.52

Note: *AUC*_24h_ = area under concentration curve, *MRT*_24h_ = mean residence time, *T*_1/2λ_ = elimination half-life, *CL*/*F* = body clearance scaled by bioavailability, *T_max_* = time of maximum concentration, *C_max_* = maximum concentration.

**Table 5:** The PK parameters of ADP and SMZ in PELF after intramuscular injection of ADP/SMZ injection at 5/25 mg/kg b.w. of SMZ in swine (*n* = 6).

Parameters	Unite	Healthy Group		Infected Group	
		ADP	SMZ	ADP	SMZ
*AUC*_24h_	h·μg/mL	49.14 ± 0.52	162.79 ± 12.45	46.94 ± 0.91	166.16 ± 12.67
*MRT*_24h_	h	6.88 ± 0.11	7.78 ± 0.17	6.67 ± 0.08	7.74 ± 0.23
*T*_1/2λ_	h	6.05 ± 0.20	8.46 ± 0.51	6.48 ± 0.62	8.29 ± 0.81
*CL*/*F*	mL/h/Kg	0.09 ± 0.02	0.14 ± 0.02	0.10 ± 0.02	0.14 ± 0.03
*T_max_*	h	1.95 ± 0.06	2.04 ± 0.08	1.96 ± 0.05	2.07 ± 0.03
*C_max_*	μg/mL	6.78 ± 0.11	25.44 ± 2.51	6.84 ± 0.25	26.35 ± 2.04

**Table 6:** The PK parameters of ADP and SMZ in plasma and PELF after intramuscular injection of ADP (5 mg/kg b.w.) and SMZ (25 mg/kg b.w.) injection in swine (*n* = 6).

Parameters	Unit	Plasma		PELF	
		ADP	SMZ	ADP	SMZ
*AUC*_24h_	h·μg/mL	7.59 ± 0.24	102.72 ± 8.25	44.82 ± 0.54	199.41 ± 10.14
*MRT*_24h_	h	7.63 ± 0.27	6.13 ± 0.12	6.43 ± 0.09	6.42 ± 0.15
*T*_1/2λ_	h	8.8 ± 0.53	4.47 ± 0.16	6.42 ± 0.43	5.63 ± 0.38
*CL*/*F*	mL/h/Kg	0.57 ± 0.03	0.24 ± 0.02	0.10 ± 0.02	0.12 ± 0.02
*T_max_*	h	1.00 ± 0.00	3.00 ± 0.00	2.00 ± 0.00	2.00 ± 0.00
*C_max_*	μg/mL	0.92 ± 0.08	12.59 ± 1.32	6.88 ± 0.14	23.4 ± 2.58

**Table 7:** The Sigmoid *E_max_* model result in PELF after intramuscular injection ADP/SMZ injection at 5/25 mg/kg b.w.

Parameters	Unite	Healthy Group		Infected Group	
		ADP	SMZ	ADP	SMZ
*E_max_*	Log_10_CFU/mL	2.07	2.3	2.15	2.36
*E*_0_	Log_10_CFU/mL	−4.78	−4.4	−4.8	−4.55
*E_max_* − *E*_0_	Log_10_CFU/mL	6.85	6.7	6.95	6.94
*EC*_50_	h	56.4	27.64	48.06	27.43
*N*	-	1.5	1.56	1.41	1.52
(*AUC*_24h_/*MIC*)*_ex_* (*E* = 0)	h	32.25	18.23	27.19	17.81
(*AUC*_24h_/*MIC*)*_ex_* (*E* = −3)	h	113.28	64.88	102.29	62.04
(*AUC*_24h_/*MIC*)*_ex_* (*E* = −4)	h	221.41	161.81	204.17	137.28

Note: (*AUC*_24h_/*MIC*)*_ex_* is ex vivo PK-PD parameters; *E* is a difference of antibacterial logarithm of lavage fluid samples incubated with drug; *E_max_* is a maximum difference of antibacterial logarithm of lavage fluid samples incubated with drug; *E*_0_ is the difference after 24 h incubation in antibacterial logarithm in control samples; *EC*_50_ is the PK-PD parameter of drug that produces 50% of the maximal antibacterial effect; *N* is the Hill coefficient which describes the steepness of the parameter-effect curve.

**Table 8:** Dosage of ADP/SMZ injection for different drug purposes.

Purpose	Healthy Group		Infected Group	
	ADP	SMZ	ADP	SMZ
Prevention (*E* = 0)	1.61 mg/kg b.w.	6.38 mg/kg b.w.	1.34 mg/kg b.w.	6.23 mg/kg b.w.
Treatment (*E* = −3)	5.66 mg/kg b.w.	22.71 mg/kg b.w.	5.06 mg/kg b.w.	21.71 mg/kg b.w.
Eradication (*E* = −4)	11.07 mg/kg b.w.	56.63 mg/kg b.w.	10.21 mg/kg b.w.	48.05 mg/kg b.w.

## Data Availability

The genome assemblies of *G. parasuis* H78 are available in the NCBI WGS database under the BioProject accession number PRJNA847363.

## Supplementary Materials

The following supporting information can be downloaded at: https://www.mdpi.com/article/10.3390/pharmaceutics14102058/s1, Table S1: Genome annotation results of *G. parasuis* H78; Table S2: Predicted virulence-associated genes identified in *G. parasuis* H78; Table S3: Predicted resistance-associated genes identified in *G. parasuis* H78.
