# Supplementary material for: Dosing Regimen of Aditoprim and Sulfamethoxazole Combination for the Glaesserella parasuis Containing Resistance and Virulence Genes
Source: Pharmaceutics. 2022 Sep 27;14(10):2058. doi: 10.3390/pharmaceutics14102058 (PMC9607282; doi:10.3390/pharmaceutics14102058)
Supplement: Supplementary file 1 [file pharmaceutics-14-02058-s001.zip › Supplementary Table S2.pdf]

Supplementary Table S2. Predicted virulence-associated genes identified in *G. parasuis* H78

Group	Name	Quantity (H78 locus_tag)	Product/ Function
Adhesion	OmpA	2 (473/2862)	Outer membrane protein OmpA and related peptidoglycan-associated (lipo) proteins
	OmpP1	2 (2806/2807)	Outer membrane protein P1
	OmpP2	2 (2998/2999)	Outer membrane protein P2
	OmpP5	2 (63/2744)	Outer membrane protein P5
	PilA	1 (928)	Tfp pilus assembly protein, major pilin PilA
	PilB	1 (929)	Tfp pilus assembly pathway, ATPase PilB
	PilC	1 (930)	Type IV pilus assembly protein PilC
	PilD	1 (932)	Tfp pilus assembly pathway, fimbrial leader peptidase PilD
	AidA	2 (2427/2434)	Pertactin family Virulence factor, outer membrane autotransporter/type V secretion pathway, adhesin AidA
	ZnuA	2 (956/1328)	Metal transporter substrate-binding protein<br>Zinc transport system substrate-binding protein
	NlpE	2 (19/3104)	Lipoprotein copper homeostasis and adhesion
	PilQ	1 (2733)	Type IV pilus secretin PilQ family protein
	PilT	1 (2823)	PilT domain-containing protein
	PilW	1 (2523)	Type IV pilus biogenesis/stability protein PilW
Hemolysin	OsmY	1 (658)	Osmotically-inducible protein OsmY
	HlyD	2 (861/2797)	Hemolysin secretion protein D
	AhpA	1 (1325)	Hemolysin regulation protein AhpA
	ShlB	1(2575)	Hemolysin activation/secretion protein

(Continued)

Supplementary Table S2. (Continued)

Group	Name	Quantity (H78 locus_tag)	Product/ Function
Hemolysin	-	2 (1607/1608)	Hemolysin or related protein, contains CBS domains
	-	6 (1966/1967/1968/1969/ 2468/2469)	Hemolysin-type calcium-binding repeat family protein
Secretion	TatC	2 (2451/2452)	Sec-independent protein secretion pathway component TatC
	TatD	3 (1247/1766/1767)	Tat protein secretion system quality control protein TatD
	CsgG	1 (1876)	Curli biogenesis system outer membrane secretion channel CsgG
	PulG	1 (2670)	Type II secretory pathway, pseudopilin PulG
	PulJ	1 (2671)	Type II secretory pathway, component PulJ
	-	1 (862)	Type I secretion system permease/ATPase
Toxin	CdtA1	1 (56/263/264)	Cytolethal distending toxin subunit A
	CdtB1	2 (54/265)	Cytolethal distending toxin B
	CdtB2	2 (53/266)	Cytolethal distending toxin protein B
	CdtB3	1 (55)	Cytolethal distending toxin subunit CdtB
	CdtC1	2 (52/267)	Cytolethal distending toxin C
	CdtC2	1 (268)	Cytolethal distending toxin protein C
	HigA	2 (375/1079)	Antitoxin component HigA of the HigAB toxin-antitoxin module
	HigB	3 (190/376/1080)	mRNA-degrading endonuclease (mRNA interferase) HigB, toxic component of the HigAB toxin-antitoxin module
	HipA	4 (2075/2076/2238/2239)	Serine/threonine protein kinase HipA, toxin component of the HipAB toxin-antitoxin module
	HipB	1 (2074)	Antitoxin HipB

(Continued)

Supplementary Table S2. (Continued)

Group	Name	Quantity (H78 locus_tag)	Product/ Function
Toxin	HicB	3 (303/1364/2783)	Antitoxin HicB
	BrnT	1 (1358)	BrnT_toxin
	BrnA	1 (1359)	BrnA_antitoxin
	TdeA	3 (2550/2551/2552)	Toxin and drug export protein A
	ToxN	3 (9/10/3094)	Abortive infection protein
	PezT	3 (41/951/1685)	Zeta toxin family protein
	MqsA	3 (69/507/2525)	Transcriptional regulator
	ChpS	2 (154/2680)	Antitoxin component of the MazEF toxin-antitoxin module
	PemK	2 (155/2679)	mRNA-degrading endonuclease, toxin component of the MazEF toxin-antitoxin module
	RelE	6 (68/374/376/508/2526/ 2563)	Addiction module toxin RelE
	RhuM	2 (2130/2835)	Virulence_RhuM
	YdaS	1 (331)	YdaS_antitoxin
	RnfH	1 (624)	Putative antitoxin component PasI (RatB) of the RatAB toxin-antitoxin module
	RatA	1 (625)	Ribosome association toxin RatA
	LktB	1 (862)	Leukotoxin translocation ATP-binding protein LktB
	YafN	1 (1449)	Antitoxin component YafN of the YafNO toxin-antitoxin module, PHD/YefM family
	Sdh5	1 (1581)	Succinate dehydrogenase flavin-adding protein, antitoxin component of the CptAB toxin-antitoxin module
	FtsN	3 (1965/1968/1969/2469)	Cell division protein FtsN; Ca2+-binding protein, RTX toxin-related

(Continued)

Supplementary Table S2. (Continued)

Group	Name	Quantity (H78 locus_tag)	Product/ Function
Toxin	VatA	19 (146/150/151/293/405/406/473/474/847/851/1849/1852/1930/1931/1932/2751/2753/2754/3061)	Virulence-associated trimeric autotransporter
	-	1 (1366)	Addiction module antitoxin
	-	1 (1367/2376)	Putative addiction module killer protein; Putative component of the toxin-antitoxin plasmid stabilization module
	-	1 (2680)	Antitoxin component of the MazEF toxin-antitoxin module
