# Supplementary material for: Dosing Regimen of Aditoprim and Sulfamethoxazole Combination for the Glaesserella parasuis Containing Resistance and Virulence Genes
Source: Pharmaceutics. 2022 Sep 27;14(10):2058. doi: 10.3390/pharmaceutics14102058 (PMC9607282; doi:10.3390/pharmaceutics14102058)
Supplement: Supplementary file 1 [file pharmaceutics-14-02058-s001.zip › Supplementary Table S3.pdf]

Supplementary Table S3. Predicted resistance-associated genes identified in *G. parasuis* H78

Group	Name	Quantity (H78 locus_tag)	Product/ Function
Antibiotic resistance	AcrA	1 (861)	Multidrug efflux pump subunit AcrA (membrane-fusion protein)
	AcrB	1 (2796)	Multidrug efflux pump subunit AcrB
	EamA	1 (152)	Multidrug transporter
	NorM	1 (2676)	Multidrug resistance protein NorM
	MexH	1 (2797)	MexH family multidrug efflux RND transporter periplasmic adaptor subunit
	-	1 (2795)	Acriflavine resistance protein, cation/multidrug efflux pump
	-	2 (239/240)	Bcr/CflA family drug resistance efflux transporter
	-	4 (456/1520/1521/2333)	Permease of the drug/metabolite transporter (DMT) superfamily
	-	8 (2123/2124/2499/2780/ 2781/2782/2841/2842)	ABC-type multidrug transport system, ATPase and permease component
Metal ion resistance	TehB	1 (1192)	Tellurite resistance methyltransferase TehB
	TerC	2 (2401/2402)	Tellurium resistance protein TerC
	MerR	1 (2086)	Mercuric resistance operon regulatory protein (Fragment)
	NlpE	2 (19/3104)	Uncharacterized lipoprotein NlpE involved in copper resistance
Other resistance	AzlC	1 (2122）	Predicted branched-chain amino acid permease (azaleucine resistance)
	-	2 (2551/2552)	RND efflux system outer membrane lipoprotein; RND superfamily resistance-nodulation-cell division antiporter
